# Augmented Reality as a Telemedicine Platform for Remote Procedural Training

**DOI:** 10.3390/s17102294

**Published:** 2017-10-10

**Authors:** Shiyao Wang, Michael Parsons, Jordan Stone-McLean, Peter Rogers, Sarah Boyd, Kristopher Hoover, Oscar Meruvia-Pastor, Minglun Gong, Andrew Smith

**Affiliations:** 1 Department of Computer Science, Memorial University of Newfoundland, St. John’s, NL A1B 3X5, Canada; 2 Faculty of Medicine, Memorial University of Newfoundland, St. John’s, NL A1B 3V6, Canada; jstonemclean@mun.ca (J.S.M.); pjrogers2001@yahoo.ca (P.R.); seb077@mun.ca (S.B.); 3 Faculty of Engineering and Applied Science, Memorial University of Newfoundland, St. John’s, NL A1B 3X5, Canada; kristopher.hoover@mun.ca

**Keywords:** telemedicine, Augmented Reality, HoloLens, ultrasound training, remote mentoring, Leap Motion sensor

## Abstract

Traditionally, rural areas in many countries are limited by a lack of access to health care due to the inherent challenges associated with recruitment and retention of healthcare professionals. Telemedicine, which uses communication technology to deliver medical services over distance, is an economical and potentially effective way to address this problem. In this research, we develop a new telepresence application using an Augmented Reality (AR) system. We explore the use of the Microsoft HoloLens to facilitate and enhance remote medical training. Intrinsic advantages of AR systems enable remote learners to perform complex medical procedures such as Point of Care Ultrasound (PoCUS) without visual interference. This research uses the HoloLens to capture the first-person view of a simulated rural emergency room (ER) through mixed reality capture (MRC) and serves as a novel telemedicine platform with remote pointing capabilities. The mentor’s hand gestures are captured using a Leap Motion and virtually displayed in the AR space of the HoloLens. To explore the feasibility of the developed platform, twelve novice medical trainees were guided by a mentor through a simulated ultrasound exploration in a trauma scenario, as part of a pilot user study. The study explores the utility of the system from the trainees, mentor, and objective observers’ perspectives and compares the findings to that of a more traditional multi-camera telemedicine solution. The results obtained provide valuable insight and guidance for the development of an AR-supported telemedicine platform.

## 1. Introduction

### 1.1. Rural Healthcare Problems

Frequently, the provision of healthcare to individuals in rural areas represents a significant logistical challenge resulting from geographic, demographic and socioeconomic factors. Recruitment and retention of healthcare providers (HCP) to rural locations continues to be a significant problem [1]. Research focused on addressing the problems associated with the provision of rural healthcare is a top priority in many countries [2].

An economical and effective solution to the lack of HCP in rural areas is telemedicine, which uses information technologies to deliver health care services over both large and small distances [2,3]. Telemedicine has many advantages, such as improved access to primary and specialized health services, improved continuity of care, increased availability of patient information, and decreased frequency of patient visits to health care specialists [4]. It also has been shown to increase patient empowerment and patient/provider satisfaction [3], while decreasing unnecessary referrals, travel and wait times, as well as the associated costs for both patients and providers [5].

### 1.2. Current Limitations

Teleconferencing is one of the main applications within telemedicine, enabling healthcare providers to interact with patients or colleagues on a regular basis. Current “talking head” interfaces used in traditional teleconferencing systems may be adequate for supporting one-on-one communication between a doctor and a patient, or even a group of doctors, but may be unsuitable in a more chaotic environment such as an emergency room (ER). Mobile robot systems have been deployed in rural settings such as “Rosie the Robot” in Nain, Labrador, Canada [6], in an attempt to address these problems; however, they remain quite expensive. Real-time consultation and support during low-frequency, high-stakes scenarios has the potential to enhance acute medical care. A system that provides a better immersive experience coupled with real-time consultation could improve performance during complex life-saving medical procedures.

### 1.3. Explosion of Computer-Mediated Reality

Google (Google Inc., Mountain View, CA, USA) released its Glass project in 2013, a technology that enabled users to connect a wearable camera and heads-up display to mobile phones via Wi-Fi. This was followed up with Google’s release of Cardboard [7], a simple cardboard box capable of transforming the ubiquitous smartphone into a Virtual Reality (VR) Head Mounted Display (HMD). Cardboard was instrumental in generating global interest and development of VR applications due to its broad appeal and accessibility. Attention then shifted to the Oculus Rift (Oculus VR, LLC, Menlo Park, CA, USA) and HTC Vive (HTC Corporation, New Taipei City, Taiwan), commercial immersive VR HMD systems connected to full computer workstations for increased performance and graphics power. More recently, Microsoft (Microsoft Corporation, Redmond, Washington, DC, USA) released the HoloLens in 2015. HoloLens was the first Augmented Reality (AR) HMD capable of spatial capture of its environment [8]. All of these products demonstrate the incremental and steady progression towards immersive AR/VR platforms and mass-market appeal. That being said, computer-mediated systems are still relatively immature, with related techniques and applications waiting to be implemented and explored.

### 1.4. Research Contributions

**Figure 1:**
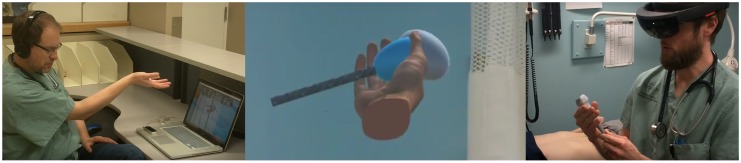
A novel Augmented Reality telemedicine platform involving real-time remote pointing and gesture capture.

In this work, we present one of the first telemedicine mentoring systems implemented using the Microsoft Hololens (Figure 1). We also demonstrate its viability and evaluated its suitability for practical use through a user study that we describe in this article. To produce the system, we tested various techniques and put them together inside the HoloLens, including: implementing a videoconference with minimal latency, relaying the holograms (3D models) of a mentor’s hands and gestures to a trainee, projecting Leap Motion (Leap Motion, Inc., San Francisco, CA, USA) recognized gestures inside the HoloLens, and allowing a mentor to control the hologram using the Leap Motion controller. The system produced was demonstrated to be viable to the degree of existing telementoring setups. In fact, we found that the Augmented Reality setup using the Hololens and Leap Motion did not show significant statistical difference when compared to the full telemedicince setup used as an experimental control. Finally, we have provided a large amount of support material and technical expertise regarding the implementation on the Hololens for the research community.

## 2. Background and Related Work

### 2.1. Augmented Reality Research in Medicine

Doctors can use AR as a visualization and mentoring aid in open surgery, endoscopy, and radiosurgery [9]. It has also commonly been used in orthopedic surgery, neurosurgery and oral maxillofacial (OMF) surgery [10], enabling the surgeon to visualize the proper positioning of their surgical instruments. AR is also useful when operating in a confined space and in close proximity to delicate and sensitive anatomical structures [11]. Many studies suggest that AR-assisted surgery appears to improve accuracy and decrease intraoperative errors in contrast to traditional, non-AR surgery [11,12,13,14,15]. However, further technological development and research is needed before AR systems can become widely adopted. General medical visualization is another task for AR to access and display types of necessary data simultaneously virtually in the surgical suite [9]. AR has the potential to support the fusion of 3D datasets of patients in real time, using non-invasive sensors like magnetic resonance imaging (MRI), computed tomography scans (CT), or ultrasound imaging. All information could then be rendered with a view of the patient at the same time, like “X-ray vision” [9]. For medical training and education, AR can play an important role [16,17]. However, gesture interaction in AR has been found to be too complicated, for both trainees and mentors [18,19].

### 2.2. Augmented Reality Research in Telemedicine

The early research mentioned in the previous section provided relevant directions and presented valuable solutions in the medical field. More advanced systems have been created as the technology has evolved. Ruzena Bajcsy et al. [20,21] collected patient’s depth maps through Microsoft Kinect and then reconstructed a virtual patient in an AR device. Using telemedicine, the mentor could then provide consultation based on the 3D model at a distance as shown in several previously developed tele-consultation applications [22,23]. However, the application required massive fund and setup [20,21,22,23]. Marina Carbone et al. [23] and Mahesh B Shenai et al. [24] created AR-assisted telemedicine applications. However, their AR system still required significant setup on both sides and had some shortcomings. It combined video from a computer-generated image and a camera-captured video, which is not as realistic as the combination of the HoloLens see-through stereoscopic vision and 3D graphics imagery. Their systems were not validated through a comparison with other more traditional telemedicine setups. Telemedicine has been proposed to solve the lack of HCP in remote locations; however, if the telemedicine application requires significant setup and even requires technical professionals in rural locations, it would lead to a new problem regarding the lack of technicians. All previous systems have this problem, while our system only requires the trainee to wear the HoloLens, which is a self-contained solution especially suitable for telemedicine. Our research attempts to overcome the limitations in previous works by designing a new telemedicine architecture using the latest telecommunication protocols and the Microsoft HoloLens. This work also provides insight into how our solution compares to more traditional telemedicine solutions.

### 2.3. Remote Collaboration

Thanks to the affordable devices described in this paper and recent communication technologies, applying Augmented and Virtual Reality to remote collaboration is a hot research topcic. Ruzena Bajcsy et al. [25] created a real-time framework to stream and receive data from Microsoft Kinect. They built a system for 3D telerehabilitation based on the framework, allowing video, audio, depth, skeleton, and other types of binary data streaming through standardized WebRTC enabled protocols. The performance of the system was tested under different network setups. John Oyekan et al. [26] also proposed a real-time remote collaboration system. The system used Microsoft Kinect to synchronously capture and transfer human activities in the task environment. It enabled the exchange of task information and digitisation of human–workpiece interactions from an expert over a network for simultaneous multi-site collaborations. Our system shares some characteristics of the systems above, such as remote collaboration support and synchronous capture and transfer of human activities for multi-site collaborations, but has been optimized to support the practical performance of a particular task (PoCUS) and has been validated for use in that context.

### 2.4. Google Glass and Microsoft HoloLens

Google Glass has been tested in a wide range of medical applications since 2014. Muensterer et al. explored its use in pediatric surgery, and concluded that Glass had some utility in the clinical setting [27]. However, substantial improvements were needed prior to actual development in the medical field related to data security and specialized medical applications [27]. Other applications include Glass being used for Disaster Telemedicine triage; however, no increase in triage accuracy was found [28]. Google Glass was used to play recorded video for mentoring purposes [29], and has also been used to address communication and education challenges in a telemedicine context [30]. Research has also explored pre-hospital care, in which Glass acted like a console for transferring patient data [31]; however, Glass could not show any advantage compared to mobile devices in this study.

Due to its novelty, research literature using the Microsoft HoloLens (released in 2015) is still scarce, especially in the medical field. Nan Cui et al. have used it in near-infrared fluorescence-based image guided surgery in which the HoloLens was used to provide vital information such as location of cancerous tissue to the surgeon [32]. Additionally, in [33], the HoloLens was used to elicit gestures for the exploration of MRI volumetric data.

### 2.5. Advantages of the HoloLens

One of the main strengths of the HoloLens as a telemedicine platform is that it is untethered—a feature valuable for chaotic environments such as the ER or operating room. It is a non-occluding AR system, in that it complements the actual scene with a relatively small amount of computer-generated imagery using a semi-transparent HMD. Furthermore, it enables a first-person view of the remote environment to be relayed and represented locally to expert observers at a remote location through a camera mounted in the middle of the HMD. Such a telepresence interface [34,35] has the potential to enhance the observers’ sense of presence, enabling them to better understand crucial circumstances and provide better guidance at critical times. From the remote learners’ perspective, the HoloLens enables recipients to participate in real-time mentoring as well as ‘just-in-time’ learning during extremely stressful situations. The ability to receive visual guidance and instructions during an infrequently performed complex medical procedure represents a significant advance for emergency personnel. A final feature is the HoloLens’ intrinsic depth-sensing and relocation ability, which can be used to support remote pointing and enhance procedural guidance. This last element is the main subject of this research.

### 2.6. Disadvantages of the HoloLens

Even though Microsoft manufactures the HoloLens with a decent 120 degrees field of view (FOV), it is still not comparable to a fully immersive HMD [36]. The weight of the HoloLens is also a problem. Discomfort and pain reports can easily be found in the literature regarding the HoloLens [36,37,38]. In addition, the ergonomics of the HoloLens are described as disappointing in various aspects, including the “airtap” gesture, weight, vision and comfort [36,37,38]. The HoloLens is also significantly lower resolution [36] than full HD monitors. Furthermore, the battery of the HoloLens can only last for approximately 100 minutes when running an application before having to be charged again. Another issue is that the HoloLens will sometimes kill an application in order to protect itself, due to the limited memory size [36]. A further limitation is that it has been designed to be exclusively as an indoor device, designed to capture its surroundings in closed environments, such as laboratories, offices and classrooms.

### 2.7. Research Focus

This research focuses on the question of how to take advantage of the HoloLens within a telemedicine AR application. The potential of AR technology has always been significant [39,40]. Even though researchers can immerse themselves nowadays in more complex virtual environments and realistic simulations, the concept of using a computer-mediated reality system in a hospital without a dedicated technician remains a hurdle as these systems are still subject to inherent technical limitations. For example, Google Glass lacked a 3D display, environment recognition ability, and had a very small field of view (FOV) to be of practical use. Since the introduction of immersive VR HMDs, such as the Oculus Rift and the HTC VIVE, VR has become more accessible as a viable option. However, these and similar devices are still tethered to workstations or have limited computing power. In this sense, the HoloLens has some particular advantages, since it has adequate computing power, does not require any tethering and does not occlude the users’ field of view. In spite of these advantages, significant efforts and multi-disciplinary cooperation are still required to assess the suitability of this and similar tools for practical use in telemedicine.

## 3. System Design

### 3.1. Prototypes

In order to test the possibility that the HoloLens can be used in the field of remote ultrasound training, we developed several prototypes covering different approaches of telecommunication technologies. These prototypes demonstrated different shortcomings, which illuminated a feasible solution to the problem. With the help of those prototypes, we proposed a final design to use of the HoloLens in a telemedicine application.

Here are the prototypes we developed in our research:

1. Gyroscope-Controlled Probe: We established a connection between an Android phone with a HoloLens using a binary communication protocol called Thrift developed by Apache. The Android application collected the orientation information of the phone and transferred it to the HoloLens application. The HoloLens application then rendered a hologram correspondingly representing a virtual ultrasound transducer. Finally, in this prototype, users can control a hologram rotating via a gyroscope located inside a mobile phone.
2. Video Conferencing: We established video conferencing between a desktop computer with a HoloLens using a local area network. Microsoft provides a built-in function called mixed reality capture (MRC) for HoloLens users. MRC enables us to capture a first-person view of the HoloLens and then present it to a remote computer. MRC is achieved by slicing the mixed reality view video into pieces and exposing those pieces through a built-in web server. Other devices can then play a smooth live video using HTTP Progressive Download. However, this could cause a noticeable latency between two ends.
3. AR together with VR: This prototype mainly remained the same structure as the previous one. The only difference was the remote player. A Virtual Reality player on a mobile phone was responsible for playing the mixed reality video. A mobile-based headset was then used to watch the VR version of the first-person view of the HoloLens. In this prototype, the mixed reality view is not a 360 degree video. Therefore, the VR user could not control the vision inside the headset, and the content was actually controlled by the HoloLens user.

Further detail about those prototypes can be found in Appendix A. An important technical aspect of the implementation is the video streaming solution we chose for use with the HoloLens. Appendix B discusses this aspect in more detail.

### 3.2. Final Design

For our final design, we took the following observations and requirements into account:

- Latency is an important factor in the quality of the teleconference experience and should be kept to a minimum.
- Verbal communication is critical for mentoring. Video conferencing within the AR without two-way voice communication was found generally less valuable.
- Immersive VR HMD for the mentors creates more challenges and requires significant technical development prior to enhancing telemedicine.
- The simplicity and familiarity of conventional technology for the mentor was an important aspect that should remain in the proposed solution.
- Remote pointing and display of hand gestures from the mentor to the trainee would be helpful for training purposes.
- Specific to ultrasound teaching, a hologram with a hand model provided additional context for remote training.

We proposed a design in order to address the requirements above through the following implementation:

1. The Leap Motion sensor was used to capture the hand and finger motion of the mentor in order to project into the AR space of the trainee.
2. Three static holograms depicting specific hand positions holding the ultrasound probe was generated and controlled by the mentor/Leap Motion.
3. MRC (video, hologram and audio) was streamed to the mentor while the mentor’s voice and hologram representations of the mentors’ hand(s) was sent to the trainee to support learning.
4. Hand model data captured by Leap Motion was serialized and bundled together with the mentor’s audio at a controlled rate to minimize latency while maintaining adequate communications.

#### 3.2.1. The Mentor’s End

We implemented an application using the Unity game engine. The final application was run on a laptop PC with a Leap Motion sensor attached to it. The hand gestures were captured and manipulated using the Leap Motion software development kit (SDK) v3.1.3. The Leap Motion SDK was used to categorize the mentor’s gestures into four different postures corresponding to four distinct holding positions present when performing PoCUS. Buttons that represent different gestures were also displayed for clicking as an alternative to compensate in case of malfunction of gesture recognition. The data from the Leap Motion was sent to the application and then serialized and compressed. We used a Logitech headphone to eliminate the presence of audio echo and to emphasize the remote sounds by keeping the surrounding noise to a minimum. The audio data from the headphone was also captured and encoded using an A-law algorithm. The computer exchanged data with the HoloLens located in a separated simulated ER (details below). The MRC video received from the HoloLens was rendered and played by a free add-on to stream video to texture using a VideoLAN Client (VLC) media backend [41].

#### 3.2.2. The Trainee’s End

**Figure 2:**
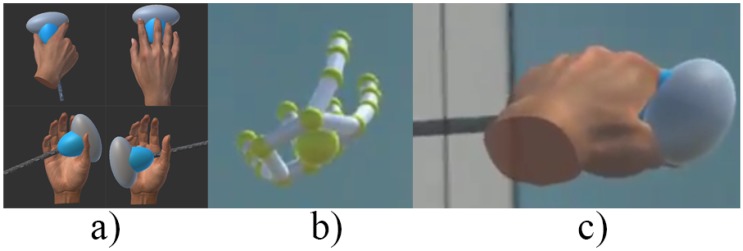
Trainee side of view: (**a**) four holograms represent different posture; (**b**) real view of the skeleton hand model conveyed by the LeapMotion on the HoloLens; (**c**) real view of one of the hand postures on the HoloLens.

We developed another application using the Unity game engine with HoloLens support. The hand models were created based on the Leap Motion Unity asset Orion v4.1.4. Several preliminary Unity 3D objects (cubes, cylinders, spheres) were combined to represent an ultrasound transducer being held in a hand model, as shown in Figure 2. The orientation and position of the hand were simulated through the data received from the mentor’s side. The audio data was decoded and played. The MRC live video was captured through Microsoft’s Device Portal representational state transfer (REST) application programming interface (API) [8].

#### 3.2.3. Settings

**Figure 3:**
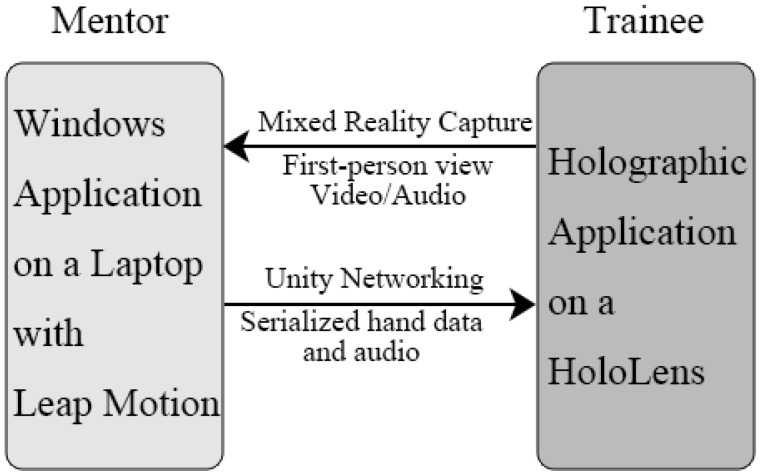
Overview of the system.

The MRC video from the trainee was captured and broadcasted by a built-in webserver running in the HoloLens. The hand data and audio data from the mentor were transmitted using Unity’s built-in multiplayer networking system called UNET. Both the HoloLens and the laptop were connected through a local network. An overview of the system is shown in Figure 3. During the experiment, the mentor and the trainee were in separate rooms to perform a simulated teleconference session.

## 4. Experimental Validation

Point of Care Ultrasound (PoCUS) represents a complex medical procedure usually performed under extremely stressful circumstances. In-person, hands-on training is highly effective; however, this remains a significant challenge for rural practitioners seeking initial training or maintenance of skill. The combination of Microsoft’s HoloLens and Leap Motion represents an AR platform capable of supporting remote procedural training. In this research, we have performed a pilot user study to explore the feasibility and user experiences of novice practitioners and a mentor using AR to enhance remote PoCUS training and compare the performance to a standard remote training platform.

### 4.1. Methods

#### 4.1.1. Participants

The ideal participants for the experiment include paramedics and undergraduate students in their first or second year who are inexperienced ultrasound users and have not participated in similar studies previously. These requirements restricted the pool of potential participants. We recruited as many individuals as possible resulting in twenty-four students from Memorial University of Newfoundland, Canada. With this amount of participants, multiple mentors could lead to a bias in the study, so we only had one mentor. This is also a compromise due to the limitation of mentor availability and time constraints.

Twelve participants with minimal PoCUS experience were enrolled in the pilot study with the HoloLens setup. Minimal experience is defined as having previously performed five or less PoCUS scans. The other twelve participants were assigned to complete the remote PoCUS training study using a “full telemedicine setup”. Further details about the reference setup used as our experimental control are introduced in the next section. Data was gathered via the same procedure and same evaluation process for baseline comparison. One mentor guided all twenty-four medical students in both setups, which helped maintain consistency of training across subjects.

#### 4.1.2. Experimental Control

We compared our solution against one of the configurations most commonly used for telemedicine today, which we refer to as the “full telemedicine setup”, and which is used as the experimental control to validate our system. This setup consists of a full overhead view of the whole patient room captured through a pan-tilt-zoom (PTZ) camera near the ceiling and a second view of the patient captured from a webcam placed on the ultrasound machine. Both cameras were live streaming together with the ultrasound screen view from the remote side to the mentor side. VSee (Vsee Lab Inc., Sunnyvale, CA, USA) was used for this secure, high-resolution and low-bandwidth video-conferencing task. Both mentor and trainees were wearing a headphone to facilitate communication.

#### 4.1.3. Procedure

Each subject was asked to complete a right upper quadrant Focused Assessment using Sonography in Trauma (FAST) ultrasound examination on a healthy volunteer under the guidance of an experienced mentor while wearing the Microsoft HoloLens in the HoloLens setup or the headphone in the full telemedicine setup. In addition to verbal guidance, the mentor provided remotely a physical demonstration of hand position and proper exploration procedures using the Leap Motion in the HoloLens setup. Performance of the trainee was independently observed and graded by a PoCUS expert using a Global Rating Scale (GRS) developed by Black et al. [42]. Participants and the mentor each completed a short Likert survey regarding the utility, simplicity and perceived usefulness of the technology. The bounds of the Likert scale measurement are 1–5, 5 for best and 1 for worst. Cognitive load was assessed using a validated instrument comprised of time to perform the task, mental effort and task difficulty rating [43]. The scale for mental effort and task difficulty ranges from 1 to 9, 1 for easiest and 9 for most difficult. Informed written consent was provided prior to participation.

#### 4.1.4. Ethics Approvals

The study design was reviewed and approved by the Human Research Ethics Authority (HREA) at Memorial University, and found to be in compliance with Memorial University’s ethics policy (HREA Code: 20161306).

#### 4.1.5. System Setup and Performance

Subjects were asked to wear the HoloLens in the HoloLens setup or the headphone in the full telemedicine prior to the start of the procedure. A curvilinear probe (1–5 Mhz) connected to a portable ultrasound (M-Turbo, Fujifilm Sonosite, Inc., Bothell, Washington, DC, USA) was used to perform the FAST examination. The ultrasound was connected to a laptop (Macbook Air, Apple Inc., Cupertino, CA, USA) via a framegrabber (AV.io; Epiphan Video, Ottawa, ON, Canada) and live-streamed over a local-area network via a standard communications software (VSee). Ultrasound streaming was both hardware and network independent from the HoloLens communications in the HoloLens setup or the telemedicine communications in the full telemedicine setup. The mentor was asked to wear a Logitech UE4500 headphone (Logitech International S.A., Lausanne, Switzerland) connected to a Microsoft Windows (Microsoft Corporation, Redmond, Washington, DC, USA) PC in both setups. A Leap Motion was attached to the PC in the HoloLens setup. The HoloLens and this PC were connected via a local-area network.

#### 4.1.6. Data and Analysis

Students and the mentor were surveyed upon completion of the task using both a short Likert survey and open-ended feedback. Cognitive load was assessed using a combination of time taken for task completion and Likert questions. Participants provided a cognitive load and task difficulty measure for each scan, and completed a general information/feedback questionnaire for the study. Data was entered into SPSS Statistics (SPSS Inc., Chicago, IL, USA) for analysis. An Independent-Samples *t*-test was used for every analysis except the completion time. The Mann–Whitney U test was used to compare task completion times.

## 5. Results

### 5.1. Trainees

As can be seen in Table 1, the feedback from the 12 participants assigned to use the HoloLens as their telemedicine tool was positive. They felt excited when using this new technology, and considered it useful for the study. Although there was a slight trend toward Full Telemedicine being superior to the HoloLens setup, there wasn’t a statistically significant difference between HoloLens and Full Setup for the questions “The technology was easy to use”, “The technology enhanced my ability to generate a suitable ultrasound image” and “The technology was overly complex”.

### 5.2. Mentor

From the mentor’s perspective, however, the technology did not reach expectations. For all categories from the mentor’s perspective, the Full Telemedicine setup was significantly superior. A detailed comparison is shown in Table 2. It is important to note that there was only one mentor, so the results have an inherent bias and cannot be generalized.

### 5.3. GRS

From the expert evaluator’s scores on the GRS for the right upper quadrant exam, there was no significant statistical difference (*p* = 0.534, *t* = 0.632, degrees of freedom (df) = 22) between the HoloLens application (2.75, standard deviation (SD) = 0.62) and the full telemedicine setup (2.91, SD = 0.67) (Table 3).

### 5.4. Completion Time, Mental Effort and Task Difficulty Ratings

We noticed that participants using the HoloLens application took much longer to finish the procedure (mean difference 153.75 s; *p* = 0.008) (Table 4) than participants completing the full telemedicine setup. The time difference between the two was statistically significant. However, trends appeared to suggest that participants felt it was easier to use the HoloLens application to perform an ultrasound scan as the mental effort rating and task difficulty rating were lower than the full setup, though there was no significant difference between the groups (Table 5).

## 6. Discussion

### 6.1. The Performance of the System

As described earlier in the Results section, there was no significant difference in overall trainee performance according to the expert evaluator. In addition, the trainee rated mental effort and task difficulty slightly lower for the HoloLens, which suggested that the HoloLens application could potentially make the task easier, though there wasn’t a statistically significant difference. However, the effectiveness of the system was rated low by the mentor. This suggests that the mentor felt it was harder to provide guidance with this setup. Furthermore, the HoloLens group took an average of 153.75 seconds longer to complete the ultrasound exploration compared to the full telemedicine group. This may be due to frequent malfunction and bad connection quality of the HoloLens. During the study, the system did not perform as well as expected.

There were several problems with the HoloLens that impacted the user experience. For example, some trainees felt that the HoloLens was too heavy and found it painful to wear. Most participants felt uncomfortable with the nose pad in particular. Contrary to what most people would expect, the nose pad of the HoloLens should not be used as a support point in a way that the weight of the device could be partially supported through it, because the device is too heavy. Instead, the HoloLens should be worn as a headband, so that the skull carries the weight of the device. Furthermore, some participants could not find a suitable fit to their head, as they had a smaller skull than the smallest fit available in the device. Even though the HoloLens has a decent field of view of 120 degrees horizontally, for many users, this is still too narrow. This is particularly relevant if we consider that the entire human field of view is slightly over 190 degrees [44,45]. This greatly influenced the user experience for all of the participants.

In the HoloLens, a stereoscopic image pair is projected to the user [46]. However, the mentor’s display is just a 2D screen without stereoscopic vision. This drawback affects the performance for remote pointing, as the mentor may lose the sense of depth. Another limitation was that the HoloLens could last for only approximately four participants or about 100 minutes before having to be charged again. One participant even had to finish the study with a connected charging cable. Another issue experienced was that the HoloLens would sometimes quit the current running application when the user was looking towards a dark area. The application would also quit when the user’s hand movements were accidentally recognized as the “bloom” gesture, which would quit the current application and open the Start menu. On the other hand, some participants enjoyed using the HoloLens. In particular, they liked how the open and non-occluding system allowed them to perform other activities while wearing the HoloLens. They were able to finish survey forms, answer their phone and talk to others without removing the device. Some participants with VR experience also mentioned that wearing the HoloLens would not cause them to get dizzy like other VR devices.

### 6.2. General Insights

Though we chose to perform the telementoring for a specific area of telemedicine (ultrasound training), most of our results have the potential to inform other applications across various disciplines and areas. We learned that, for building a communication application, the quality of connection (latency) would be the first problem noticed by an operator [47,48]. During the experiment, we noticed that traditional user interfaces such as buttons and keyboards were more reliable compared to new ones such as gesture and speech. For inexperienced users, if the new user interfaces worked improperly only one or two times, they may abandon them. The HoloLens still has some limitations and is not yet ready for practical application. However, the idea of presenting 3D objects in addition to one’s vision may still be beneficial in various scenarios such as virtual fitting dressing rooms, remote presenting and remote teaching. We also learned that the performance is not always improved with new technology, as this AR setup did not show a statistical difference when compared to a low cost setup. On the other hand, these results are not negative either, and can only improve as the technology advances, suggesting that these types of AR systems have the potential to become a helpful tool in telemedicine, just like the full telemedicine set-up, provided we can make them faster, more robust and lightweight.

### 6.3. Limitations

There were many limitations to this pilot study. First of all, the experiment was not under entirely real circumstances, as the connection was established under a local network. The reason for using a local network was to provide a dedicated bandwidth and not rely on the variability of the university local area network, which was important to support the high bandwidth requirements of the application. Another limitation would be the technical problems that happened in the testing environment. Next, every participant reported different levels of discomfort with the HoloLens, which negatively impacted the experience. In addition, only one mentor was involved in the study, so the mentor gradually familiarized himself with the whole setup, which may have caused an increasing trend in performance across trials due to the learning effect. Finally, the results could be biased due to a low sample size. Time and budget limitations forced us to have a small study. Future studies could measure performance using only one assessment, which might save a substantial amount of time.

During the study, the mentor was able to indicate desired hand position through the Leap Motion sensing technology. After five participants used the system, however, Leap Motion appeared to be working improperly. It was unable to recognize and provide the orientation of the hand correctly. It is still unknown why this occurred. However, when we unplugged the Leap Motion sensor for a while, the problem could be solved. The study was then paused until the Leap Motion was working correctly again. For the next study, we plan to have multiple Leap Motion sensors to avoid this issue.

Most participants also found it difficult to locate the holograms (3D models). We put hand models at a fixed position and enabled the mentors to reset it to the trainee’s current field of vision remotely. The trainee could also reset it by voice command. However, when a participant could not find the model, often times the participant would move their head rapidly in order to locate the model. This behaviour made the reset task even more difficult for the mentors. Additionally, the audio data was not streamed from the mentor to the HoloLens. Normally, a network connection will be created between two sides of network users, and network data will be sent byte by byte quickly. This is network streaming, which is considered a good way to transfer data. However, in our system, the audio was sent progressively after a short period. This may have required more bandwidth and led to a higher latency. The reason was that the streaming function is not provided by the UNET system. Instead, an internal buffer was included in UNET to secure reliable sequenced transmission. With all of these restrictions, the bandwidth requirement for our system reached slightly higher than 50 Mbps, with almost 50 Mbps for mixed reality video (480 p, 940 × 480, 15 Hz) transmission, 96 kbps for audio and 5 bps for hand data. This video quality was lower than the full telemedicine setup, which provided 720 p using VSee. We believe that the latency and the quality should be considerably improved if we create a network streaming with better protocol and hardware environment. Microsoft just released a project called Mixed Remote View Compositor, which provides the ability to incorporate near real-time viewing of mixed reality view. It is achieved through low-level Media Foundation components, which tends to resolve the MRC latency problem with Dynamic Adaptive Streaming over HTTP (DASH), as discussed in the next section.

### 6.4. Privacy

Most patients are willing to have their doctor use face-mounted wearable computers, even when unfamiliar with the technology [49]. However, some patients have expressed concerns about privacy, which can certainly be a concern when a camera is pointing directly at them [49]. In this research, we serialized hand and audio data prior to network transmission. Compression and encryption can also be added into the serialization process. Furthermore, MRC is protected by a username and password combination. This setup provides a basic protection to the patient’s privacy. However, all of the data is transmitted through the Internet, which may make it vulnerable to hackers. The privacy regarding recording is also another concern when a videoconference is established.

### 6.5. Future Work

In the user study, we noticed that the quality of the connection. In particular, the latency, was the key reason for poor performance. The latency came from two sides.

First, the audio data was progressively transferred together with the hand data from the mentor to the HoloLens instead of streaming. We believe that the latency should be considerably improved if we create a network streaming with better protocol and hardware environment. Microsoft released a Sharing server in their HoloToolkit project on Github.com. It allows applications to span multiple devices, and enables collaboration between remote users. The server runs on any platform, and can work with any programming language.

Second, the built-in Mixed Reality Capture (MRC) function is achieved by HTTP progressive download. The mixed reality view is continuously being recorded for a short period of time into a series of video files, and then exposed on the built-in web server (also known as the Device Portal) on the HoloLens. After that, other applications can then access the web server, download and then play the recorded serial video files progressively. This method is suitable for live broadcast applications, but inappropriate for an application with instant communication requirements. Microsoft just released a project called Mixed Remote View Compositor, which provides the ability to incorporate near real-time viewing of mixed reality view. It is achieved through low level Media Foundation components, which tends to resolve the MRC latency problem with Dynamic Adaptive Streaming over HTTP (DASH).

With the help of these projects, we redesigned the whole networking connections, and preliminarily reduced the latency from 2–3 s to less than 500 ms. The bandwidth requirement for this design is also potentially reduced to 4 Mbps, which suggests the possibility to run this system under the LTE network.

**Figure 4:**
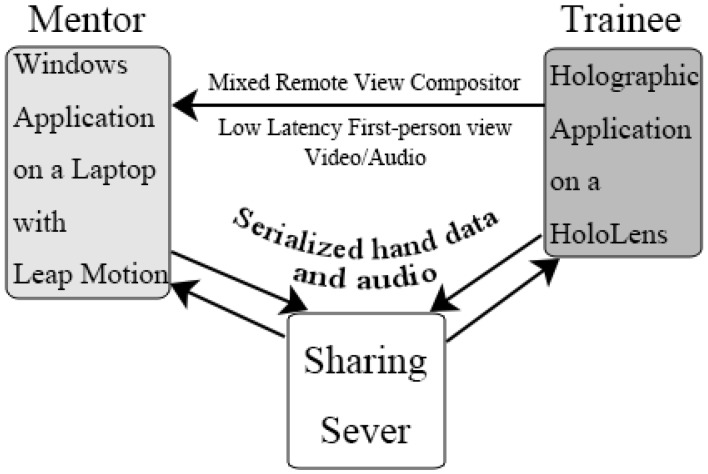
Overview of our suggestions for an improved system.

The way to present the hand model could also be changed. The hologram with a hand model would be presented right in the middle of the users’ vision. Together with the latency, this improved version could improve the user experience. Figure 4 shows the pipeline of our proposal for an improved system.

The expectation that the new system could yield better results is simply a hypothesis by our team at this time. We believe that reducing the delay in the communication between mentors and trainees to a maximum is very important to the viability of the system. However, the software projects involved in the preliminary improvements are experimental Github projects released by Microsoft just recently. Currently, all of these projects have high update rates and quite a few bugs. Not even their executability can be guaranteed. For research purposes, projects should use at least an alpha release for a user study to produce results that are stable and convincing. Therefore, we believe that this improved system might be suitable for a future study if a stable version is produced. In this research, we performed the user study using a stable system. In order to evaluate the effect of the suggested prototype improvements in a way that is reliable and convincing, a new user study with a larger number of participants and mentors would be the appropriate way to continue this work.

## 7. Conclusions

We have presented the design and implementation of an ultrasound telementoring application using the Microsoft HoloLens. Compared to available telementoring applications that mostly include visual and auditory instructions, the system introduced here is more immersive as it presents a controlled hand model with an attached ultrasound transducer. Compared to other gesture based AR systems, our system is easier to set up and run. The trainee is wearing an AR headset and following voice instructions together with the mentor’s transported hand gestures. The pilot user study with 12 inexperienced sonographers (medical school students) demonstrated that this could become an alternative system to perform ultrasound training. However, the HoloLens still needs to be improved, as every participant reported different levels of physical discomfort during the study, and an assistant must ensure that the device is properly worn. Furthermore, the completion time for the HoloLens application is longer than the other setup. Finally, the single mentor reported that the task became harder when using the HoloLens. A new system with significant improvements has the potential to be a feasible telemedicine tool, and we plan to evaluate this with a full user study in the near future. Other applications that could be studied in future research include other training systems and exploratory adventures in uncharted territories, such as creating an interactive social network application on the HoloLens.

### 7.1. Main Contributions of this Research

There are several components involved in this research, exploring the possibilities in different directions. The main contributions of this research are shown below:

- We have developed one of the first telemedicine mentoring systems using the Microsoft Hololens. We then demonstrated its viability and evaluated its suitability in practical use through a user study.
- We have tested various techniques and put them together inside the HoloLens, including: overlaying the holograms; controlling the hologram using a smart phone; implementing a videoconference with minimal latency; projecting Leap Motion recognized gestures inside the HoloLens. All of these attempts are meaningful and useful for HoloLens-related developers due to its novelty.
- We have found that the performance of the AR setup using the Hololens and Leap Motion did not show significant statistical difference when compared to a full telemedicine setup, demonstrating the viability of the system.
- Until August 2017, the documentation about HoloLens development is still scarce. When planning to develop a new application under the HoloLens, lack of support is currently a primary problem. We have provided a large amount of support material to follow up on this work, which could be considered a valuable asset for the research community.

Above all, the most difficult part of this research was clearly the implementation of the hand-shape hologram control part. We had to gather the recognized hand data from the Leap Motion controller, serialize and transfer it to the HoloLens side, and then interpret the received serialized data into a hand-shape hologram. All of this was done with very little documentation available. After that, merging this part together with Microsoft’s Github projects was also instrumental for finally completing this work.

## Figures and Tables

**Table 1:** Trainee’s opinions on the efficacy and difficulty of the HoloLens and Full Telemedicine Set-Up.

	HoloLens Score Out of 5 (Standard Deviation)	Full Telemedicine Set-Up Score Out of 5 (Standard Deviation)	*p*-Value	*t*-Value	Degree of Freedom
The technology was easy to setup and use	4.08(0.90)	4.67(0.49)	0.065	1.969	17.039
The technology enhanced my ability to generate a suitable ultrasound image	4.50(0.67)	4.58(0.51)	0.737	0.340	22
The technology was overly complex	1.92(0.79)	1.42(0.51)	0.081	−1.832	22

**Table 2:** Mentor’s opinions on the efficacy and real-life application of the HoloLens and Full Telemedicine Set-Up.

	HoloLens Score Out of 5 (Standard Deviation)	Full Telemedicine Set-Up Score Out of 5 (Standard Deviation)	*p*-Value	*t*-Value	Degree of Freedom
I was able to telementor the student effectively	2.92(1.00)	3.67(0.65)	0.04	2.183	22
The technology was effective in enhancing remote ultrasound training	2.50(1.17)	3.75(0.45)	0.004	3.458	14.227
I would be able to mentor a trainee in a real-life stressful situation with this technology	2.25(1.14)	3.42(0.67)	0.007	3.062	17.783

**Table 3:** Global Rating Scale for right upper quadrant exam of the HoloLens and Full Telemedicine Set-Up.

	HoloLens Score Out of 5 (Standard Deviation)	Full Telemedicine Set-Up Score Out of 5 (Standard Deviation)	*p*-Value	*t*-Value	Degree of Freedom
Preparation for Procedure	2.92(0.79)	3.00(0.60)	0.775	0.290	22
Patient Interaction	3.00(0.43)	3.08(0.51)	0.670	0.432	22
Image Optimization	3.00(0.60)	3.08(0.51)	0.719	0.364	22
Probe Technique	2.83(0.58)	2.83(0.72)	1.000	0.000	22
Overall Performance	2.75(0.62)	2.91(0.67)	0.534	0.632	22

**Table 4:** Trainee’s completion time for the HoloLens and Full Telemedicine Set-Up.

	HoloLens Score (Standard Deviation)	Full Telemedicine Set-Up Score (Standard Deviation)	*p*-Value
Completion Time (Seconds)	536.00(142.11)	382.25(124.09)	0.008

**Table 5:** Trainee’s perception of mental effort and task difficulty for the HoloLens and Full Telemedicine Set-Up.

	HoloLens Score (Standard Deviation)	Full Telemedicine Set-Up Score (Standard Deviation)	*p*-Value	*t*-Value	Degree of Freedom
Mental Effort Score out of 9	3.83(1.59)	4.58(1.73)	0.280	1.107	22
Task Difficulty Score out of 9	3.42(1.31)	4.25(1.66)	0.186	1.365	22

**Figure A1:**
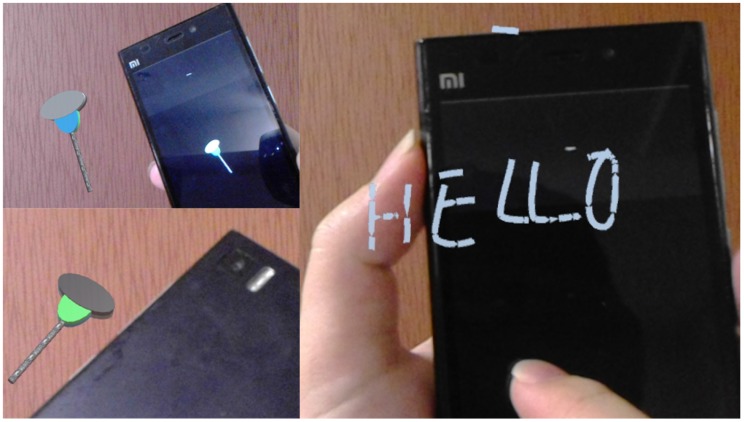
Virtual Probe controlled by the gyroscope located in the mobile phone. Remote drawing can also be achieved by drawing on the screen of the phone.

## Supplementary Materials

- A video about this study is available online at http://www.wsycarlos.com/teleholo_video.html.
- To provide an overview of the lessons learned in this research, the advantages and disadvantages of the different prototypes attempted to reach our proposed solution are illustrated in Appendix A.
- Specific technical details about the video streaming solutions explored for the HoloLens are discussed in Appendix B.
- Source code of the whole project can be accessed via https://bitbucket.org/wsycarlos/mrcleaphand.

There are several projects and plugins involved in the implementation of our study. They played an important role in the implementation of this research:

- Mixed Remote View Compositor in HoloLensCompanionKit:
  https://github.com/Microsoft/HoloLensCompanionKit/tree/master/MixedRemoteViewCompositor
- Sharing Sever in HoloToolkit:
  https://github.com/Microsoft/HoloToolkit/tree/master/Sharing
  https://github.com/Microsoft/HoloToolkit-Unity
- Leap Motion for Unity Development
  https://developer.leapmotion.com/unity
- AVPro Video plugin developed by RenderHeads
  http://renderheads.com/product/avpro-video/

## Appendix A. Development of a Telemedicine Prototype Using the Hololens

### Appendix A.1. Gyroscope-Controlled Probe

In order to test the possibility that the HoloLens can be used in the field of remote ultrasound training, we developed an initial prototype simulating a virtual ultrasound transducer on the HoloLens with its orientation controlled by the gyroscope inside a mobile phone (Figure A1). A hologram of an ultrasound transducer was projected within the trainee’s field of view while the gyroscope was accessed in an Android phone. The orientation information of the phone was live-streamed to a local HoloLens. The orientation data enabled the hologram to be adjusted accordingly. The basic objective was to demonstrate that a mentor could represent a motion or gesture in the HoloLens AR space and provide user feedback.

This early-stage prototype was deployed on the HoloLens with 10 participants agreeing to do a pilot test of the application. The research protocol involving human subjects for this and other related trials was reviewed and approved by the Health Research Ethics Authority in St. John’s, Newfoundland and Labrador. Each participant used the system for five minutes prior to providing general feedback. One participant indicated that “having virtual objects around the actual environment is so cool”. Most people felt they were able to gain some additional information without extra effort. However, one concern highlighted the challenges associated with how the trainees should actually hold the ultrasound probe. This resulted in the addition of a hand model to the virtual transducer. Other feedback highlighted the importance of two-way communications, ability to manipulate the probe in 3D space (as opposed to simply roll, pitch, yaw), and the importance of capturing hand as well as probe motion.

### Appendix A.2. Video Conferencing

Feedback from the first prototype prompted us to consider a video conferencing application between the HoloLens and a desktop computer. Microsoft provides a built-in function called mixed reality capture (MRC) for developers. The HoloLens can create an experience of mixing the real and digital worlds, with the MRC becoming a valuable tool to capture this experience from a first-person point of view. The lack of compatibility between the HoloLens and video streaming protocols is the chief obstacle of this video conferencing task. All immersive apps built for the HoloLens run on the Universal Windows Platform (UWP) and hence are required to be built with the Unity Engine. Unity owns the “Asset Store”, which contains many free and paid plugins. However, the HoloLens is a new product with limited access and no related video plugins available in the Asset Store yet. Finally, after several failed attempts (more detail in Appendix B), a plugin developed by RenderHeads (RenderHeads Ltd, Liverpool, UK), called AVPro Video was located. AVPro Video provides powerful video playback solutions on various platforms including the HoloLens.

The team created a video conference in the lab using a local area network and again sought user feedback. During this iteration, the participants had difficulty focusing on performing the ultrasound procedure with a video feed streaming in their field of view. It was deemed uncomfortable to have both a video and a dynamic probe hologram simultaneously. Furthermore, the latency of the live-stream video, which could reach as high as 10 seconds, was unacceptable. On the other hand, attempting to use an MRC tool would cause the system to reduce the rendering to 30 Hz, and would also cause the hologram content in the right eye of the device to “sparkle” [8,50], which would be an undesirable artifact. The HoloLens was unable to maintain sufficient rendering quality with the MRC enabled, subsequently explaining the increased latency during a video decoding task. For this reason, the team concluded that video conferencing was not a suitable choice for the HoloLens and its Holographic Processing Unit (HPU) at the time.

### Appendix A.3. AR Together with VR

Armed with new knowledge and experience learned from previous attempts, we hypothesized that the 3D appearance of the remote environment could be captured by the HoloLens and represented locally to an expert observer wearing an immersive VR HMD (the Oculus Rift). Such an immersive telepresence interface [34,35] had the potential to enhance the mentors’ sense of presence. The MRC live video was broadcasted from the HoloLens’ first-person perspective through a built-in web server on the HoloLens. The Wowza Streaming Engine [51] was chosen to re-broadcast the MRC video using the Real Time Streaming Protocol (RTSP). MRC video was transferred and played to the mentor using the VR headset, recreating the first person view for the mentor in an attempt to provide a high sense of telepresence.

Feedback from this system pertained mainly to the mentor’s experience. The expectation was that the mentor, wearing the VR headset, would be able to adjust their view by moving their head. In this iteration, the VR headset simply acted as a monitor displaying the HoloLens’ perspective. This resulted in the remote trainee being the individual controlling the expert’s view while requiring additional guidance on where to look. Anecdotally, this appeared to increase the cognitive load on both the trainee and the mentor and occasionally triggered VR sickness symptoms due to the visually-induced perception of self-motion. Finally, the mentor indicated that he was significantly more comfortable with more traditional input methods such as mouse, keyboard and touch rather than a more modern user interface such as voice and gesture.

## Appendix B. Video Streaming on the HoloLens

Prior to the study, when we tried to implement a videoconferencing application on the HoloLens, several streaming protocols were tested. All holographic applications built for the HoloLens run on the Universal Windows Platform (UWP). The Unity Engine was the default option for this task. The Unity Engine is mainly designed to develop games, and lacked adequate support related to video playback for the HoloLens. The “Asset Store” contains many plugins that can do this job. However, the HoloLens is a new product with limited access and most video plugins cannot work properly on the HoloLens. The compatibility of the HoloLens was becoming a problem for this videoconferencing task. We performed several experiments to find a best solution. Some details are listed below.

### Appendix B.1. Web Real-Time Communication (WebRTC)

Web Real-Time Communication (WebRTC) is the latest protocol for real-time video streaming, but it is still an experimental project. It is fast, low latency and compatible with the newest browsers. The problem was how to integrate it into Unity. We tried different ways to make it work inside Unity but failed. The first attempt was to use a WebGL build in Unity. WebGL is still a project in development, and is not compatible with the HoloLens. The performance of this implementation was not quite good—an empty scene with a cube required more than 20 seconds to load on a new iPhone and at a frame rate no better than 15 frames per second (fps). The second thought was to embed a browser (web-view) inside a Net application. However, it was hard to find a good framework. Several frameworks were written for web-view function, but either were not compatible with HTML5 elements or only worked on Unity Windows PC.

### Appendix B.2. HTTP Live Streaming (HLS)

HTTP Live Streaming (HLS) was the first protocol that worked in our experiments. We could use either Open Broadcaster Software (OBS) or ffmpeg (video encoding/decoding library) to capture the stream for HLS. A Nginx web server and nginx-rtmp-module with HLS support was used as the server. It is fast, smooth, high-resolution, compatible with almost every platform (works perfect on Windows, OSX, iOS, Android, HoloLens as well as a browser). The problem was the prior delay for every stream because it needs to slice the video into small parts. If the parts are too small, it would require more CPU usage, while, if the parts are large, then the latency would become quite high.

### Appendix B.3. Real-Time Messaging Protocol (RTMP) and Real-Time Streaming Protocol (RTSP)

Real-Time Messaging Protocol (RTMP) and Real-Time Streaming Protocol (RTSP) were the next two we tried. Open Broadcaster Software(OBS) or ffmpeg (video encoding/decoding library) were also suitable for capturing the stream for RTMP and RTSP. A Nginx web server and nginx-rtmp-module could also be used as the server. They were fast, smooth, high-resolution and low latency. The problem was the compatibility. For RTMP, it required a flash player at the client side, which made it inaccessible to many platforms, and hard to integrate into Unity. We had tried to use FluorineFx, a framework made RTMP compatible with Microsoft Net. It was outdated with a lot of errors—not very helpful. For RTSP, some plugins provided the support to play it under the Unity environment. However, most of them were not designed for the HoloLens.

We finally found a plugin developed by RenderHeads called AVPro Video, which provided powerful video playback solutions on various platforms including the HoloLens. HTTP Progessive Streaming, HLS and RTSP were all supported on the HoloLens. With RTSP protocol, the latency caused by the video streaming was eliminated. However, the latency caused by the Mixed Reality Capture was still significant in video conferencing.

### Appendix B.4. Dynamic Streaming over HTTP (DASH)

Media Foundation is a video engine developed by Microsoft. It is designed to provide video playback support for the Universal Windows Platform (UWP). This is the native video support for the HoloLens. We could add playback of adaptive streaming multimedia content to UWP apps using Media Foundation. This feature currently supports playback of HTTP Live Streaming (HLS) and Dynamic Streaming over HTTP (DASH) content. For HLS, the latency is the problem. Therefore, DASH was chosen in the end. The Mixed Remote View Compositor project released by Microsoft is used in our upgraded version, which provides the ability to incorporate near real-time viewing of mixed reality view. It is achieved through low level Media Foundation components. The video feed is captured through the camera on the HoloLens, and then mixed with the virtual 3D objects. After that, the DASH protocol is used to stream the final mixed reality video. With all these efforts, a low latency mixed reality playback is finally achieved.

## References

[1] Dussault G., Franceschini M.C. (2006). Not enough there, too many here: Understanding geographical imbalances in the distribution of the health workforce. Hum. Resour. Health.

[2] Rural Health in Rural Hands: Strategic Directions for Rural, Remote, Northern and Aboriginal Communities. http://www.ruralontarioinstitute.ca/file.aspx?id=29b5ba0b-c6ce-489f-bb07-2febfb576daa.

[3] Aarnio P., Rudenberg H., Ellonen M., Jaatinen P. (2000). User satisfaction with teleconsultations for surgery. J. Telemed. Telecare.

[4] Moehr J., Schaafsma J., Anglin C., Pantazi S., Grimm N., Anglin S. (2006). Success factors for telehealth: A case study. Int. J. Med. Inform..

[5] Doze S., Simpson J., Hailey D., Jacobs P. (1999). Evaluation of a telepsychiatry pilot project. J. Telemed. Telecare.

[6] Mendez I., Jong M., Keays-White D., Turner G. (2013). The use of remote presence for health care delivery in a northern Inuit community: A feasibility study. Int. J. Circumpolar Health.

[7] Facebook Has Oculus, Google Has Cardboard. https://www.cnet.com/news/facebook-has-oculus-google-has-cardboard/.

[8] Taylor A.G. (2016). Develop Microsoft HoloLens Apps Now.

[9] Riva G., Grimshaw M. (2014). Medical Clinical Uses of Virtual Worlds. The Oxford Handbook of Virtuality.

[10] Müller S., Maier-Hein L., Mehrabi A., Pianka F., Rietdorf U., Wolf I., Grenacher L., Richter G., Gutt C., Schmidt J. (2007). Creation and establishment of a respiratory liver motion simulator for liver interventions. Med. Phys..

[11] Ogata T., Onuki J., Takahashi K., Fujimoto T. (2005). The use of computer-assisted system in ear surgery. Oto-Rhino-Laryngol. Tokyo.

[12] Meola A., Cutolo F., Carbone M., Cagnazzo F., Ferrari M., Ferrari V. (2016). Augmented reality in neurosurgery: A systematic review. Neurosurgical rev..

[13] Bly R.A., Chang S.H., Cudejkova M., Liu J.J., Moe K.S. (2013). Computer-guided orbital reconstruction to improve outcomes. JAMA Facial Plast. Surg..

[14] Qu M., Hou Y., Xu Y., Shen C., Zhu M., Xie L., Wang H., Zhang Y., Chai G. (2015). Precise positioning of an intraoral distractor using augmented reality in patients with hemifacial microsomia. J. Cranio-Maxillofac. Surg..

[15] Badiali G., Ferrari V., Cutolo F., Freschi C., Caramella D., Bianchi A., Marchetti C. (2014). Augmented reality as an aid in maxillofacial surgery: Validation of a wearable system allowing maxillary repositioning. J. Cranio-Maxillofac. Surg..

[16] Herron J. (2016). Augmented Reality in Medical Education and Training. J. Electron. Resour. Med. Lib..

[17] Foronda C.L., Alfes C.M., Dev P., Kleinheksel A., Nelson Jr D.A., O’Donnell J.M., Samosky J.T. (2017). Virtually Nursing: Emerging Technologies in Nursing Education. Nurse Educ..

[18] Alem L., Tecchia F., Huang W. (2011). HandsOnVideo: Towards a gesture based mobile AR system for remote collaboration. Recent Trends of Mobile Collaborative Augmented Reality Systems.

[19] Tecchia F., Alem L., Huang W. 3D helping hands: a gesture based MR system for remote collaboration. Proceedings of the 11th ACM SIGGRAPH International Conference on Virtual-Reality Continuum and Its Applications in Industry.

[20] Anton D., Kurillo G., Yang A.Y., Bajcsy R. Augmented Telemedicine Platform for Real-Time Remote Medical Consultation. Proceedings of the International Conference on Multimedia Modeling.

[21] Kurillo G., Yang A.Y., Shia V., Bair A., Bajcsy R. New Emergency Medicine Paradigm via Augmented Telemedicine. Proceedings of the International Conference on Virtual, Augmented and Mixed Reality.

[22] Riva G., Dakanalis A., Mantovani F., Sundar S.S. (2015). Leveraging Psychology of Virtual Body for Health and Wellness. The Handbook of the Psychology of Communication Technology.

[23] Carbone M., Freschi C., Mascioli S., Ferrari V., Ferrari M. A Wearable Augmented Reality Platform for Telemedicine. Proceedings of the International Conference on Virtual, Augmented and Mixed Reality.

[24] Shenai M.B., Dillavou M., Shum C., Ross D., Tubbs R.S., Shih A., Guthrie B.L. (2011). Virtual interactive presence and augmented reality (VIPAR) for remote surgical assistance. Oper. Neurosurg..

[25] Antón D., Kurillo G., Goñi A., Illarramendi A., Bajcsy R. (2017). Real-time communication for Kinect-based telerehabilitation. Future Gener. Comput. Syst..

[26] Oyekan J., Prabhu V., Tiwari A., Baskaran V., Burgess M., Mcnally R. (2017). Remote real-time collaboration through synchronous exchange of digitised human–workpiece interactions. Future Gener. Comput. Syst..

[27] Muensterer O., Lacher M., Zoeller C., Bronstein M., Kübler J. (2014). Google Glass in pediatric surgery: An exploratory study. Int. J. Surg..

[28] Cicero M.X., Walsh B., Solad Y., Whitfill T., Paesano G., Kim K., Baum C.R., Cone D.C. (2015). Do you see what I see? Insights from using google glass for disaster telemedicine triage. Prehospital Disaster Med..

[29] Knight H., Gajendragadkar P., Bokhari A. (2015). Wearable technology: Using Google Glass as a teaching tool. BMJ Case Rep..

[30] Moshtaghi O., Kelley K., Armstrong W., Ghavami Y., Gu J., Djalilian H. (2015). Using Google Glass to solve communication and surgical education challenges in the operating room. Laryngoscope.

[31] Widmer A., Müller H. (2014). Using Google Glass to enhance pre-hospital care. Swiss Med. Inf..

[32] Cui N., Kharel P., Gruev V. (2017). Augmented reality with Microsoft HoloLens Holograms for Near Infrared Fluorescence Based Image Guided Surgery. Proc. SPIE.

[33] Pham T., Tang A. User-Defined Gestures for Holographic Medical Analytics. Proceedings of the Graphics Interface.

[34] Beck S., Kunert A., Kulik A., Froehlich B. (2013). Immersive group-to-group telepresence. IEEE Trans. Vis. Comput. Graph..

[35] Zhang C., Cai Q., Chou P., Zhang Z., Martin-Brualla R. (2013). Viewport: A distributed, immersive teleconferencing system with infrared dot pattern. IEEE Trans. Multimedia.

[36] Hachman M. We Found 7 Critical HoloLens Details That Microsoft Hid Inside Its Developer Docs. http://www.pcworld.com/article/3039822/consumer-electronics/we-found-7-critical-hololens-details-that-microsoft-hid-inside-its-developer-docs.html.

[37] Looker J., Garvey T. Reaching for Holograms. http://www.dbpia.co.kr/Journal/ArticleDetail/NODE06588268.

[38] Munz G. Microsoft Hololens May Cause Discomfort as It Gets Extremely Hot. https://infinityleap.com/microsoft-hololens-may-cause-discomfort-as-it-gets-extremely-hot/.

[39] Shapiro A., McDonald G. (1992). I’m not a real doctor, but I play one in virtual reality: Implications of virtual reality for judgments about reality. J. Commun..

[40] Kaltenborn K., Rienhoff O. (1993). Virtual reality in medicine. Methods Inf. Med..

[41] De Lattre A., Bilien J., Daoud A., Stenac C., Cellerier A., Saman J.P. VideoLAN Streaming Howto. https://pdfs.semanticscholar.org/5124/351d69bd3cbd95eca1e282fb8da05cd3761c.pdf.

[42] Black H., Sheppard G., Metcalfe B., Stone-McLean J., McCarthy H., Dubrowski A. (2016). Expert facilitated development of an objective assessment tool for point-of-care ultrasound performance in undergraduate medical education. Cureus.

[43] DeLeeuw K.E., Mayer R.E. (2008). A comparison of three measures of cognitive load: Evidence for separable measures of intrinsic, extraneous, and germane load. J. Educ. Psychol..

[44] Pourazar B., Meruvia-Pastor O. (2016). A Comprehensive Framework for Evaluation of Stereo Correspondence Solutions in Immersive Augmented and Virtual Realities. J. Virtual Reality Broadcast..

[45] Howard I.P., Rogers B.J. (1995). Binocular vision and stereopsis.

[46] Staff C. (2017). Address the consequences of AI in advance. Commun. ACM.

[47] Anvari M., Broderick T., Stein H., Chapman T., Ghodoussi M., Birch D.W., Mckinley C., Trudeau P., Dutta S., Goldsmith C.H. (2005). The impact of latency on surgical precision and task completion during robotic-assisted remote telepresence surgery. Comput. Aided Surg..

[48] Geelhoed E., Parker A., Williams D.J., Groen M. Effects of Latency on Telepresence. http://shiftleft.com/mirrors/www.hpl.hp.com/techreports/2009/HPL-2009-120.pdf.

[49] Prochaska M.T., Press V.G., Meltzer D.O., Arora V.M. (2016). Patient Perceptions of Wearable Face-Mounted Computing Technology and the Effect on the Doctor-Patient Relationship. Appl. Clin. Inf..

[50] Microsoft Mixed Reality Capture for Developers. https://developer.microsoft.com/en-us/windows/mixed-reality/mixed_reality_capture_for_developers.

[51] Aloman A., Ispas A., Ciotîrnae P., Sanchez-Iborra R., Cano M.D. Performance evaluation of video streaming using MPEG DASH, RTSP, and RTMP in mobile networks. Proceedings of the IFIP Wireless and Mobile Networking Conference (WMNC).

